# Camel milk consumption is associated with less childhood stunting and underweight than bovine milk in rural pastoral districts of Somali, Ethiopia: a cross-sectional study

**DOI:** 10.1017/jns.2021.75

**Published:** 2021-09-20

**Authors:** Anbissa Muleta, Dejene Hailu, Barbara J. Stoecker, Tefera Belachew

**Affiliations:** 1 Department of Food Science and Nutrition, Jigjiga University, Jigjiga, Ethiopia; 2 School of Nutrition, Food Science and Technology, Hawassa University, Hawassa, Ethiopia; 3 Department of Public and Environmental Health, College of Health Sciences, Hawassa University, Hawassa, Ethiopia; 4 Department of Nutritional Sciences, Oklahoma State University, Stillwater, OK, USA; 5 Department of Nutrition and Dietetics, Jimma University, Jimma, Ethiopia

**Keywords:** Bovine milk, Camel milk, Growth failures, Pre-schoolers, AOR, adjusted odds ratio, BM, bovine milk, CaM, camel milk, CI, confidence interval, Hb, haemoglobin, SSA, sub-Saharan Afric, UNICEF, United Nations Children's Fund, VIF, variance inflation factor, WHO, World Health Organization

## Abstract

Undernutrition is a major global health problem. Various types of animal milk are used for feeding children at early ages; however, associations of camel milk (CaM) and bovine milk (BM) with the nutritional status of children have not been explored. A comparative community-based cross-sectional study was conducted among pre-schoolers in rural pastoral districts of Somali, Ethiopia. Children were selected from households with lactating camels or cows. Anthropometric measurements followed standard procedures for height-for-age, weight-for-age and weight-for-height scores. Independent sample *t*-tests identified significant differences in anthropometric indices based on the type of milk consumed. Multivariable logistic regression was used to examine associations between milk consumption and other predictors of growth failures. The prevalence of stunting was 24⋅1 % [95 % confidence interval (CI) 20⋅5, 28⋅3] of pre-schoolers, 34⋅8 % (95 % CI 29⋅9, 39⋅6) were wasted and 34⋅7 % (95 % CI 30⋅1, 39⋅9) were underweight. Higher proportions of BM-fed children were severely stunted, wasted and underweight compared with CaM consumers. Using logistic regression models, children who consumed BM [adjusted odds ratio (AOR): 2⋅10; 95 % CI 1⋅22, 3⋅61] and who were anaemic (AOR: 4⋅22; 95 % CI 2⋅23, 7⋅98) were more likely to be stunted than their counterparts, while girls were less likely to be stunted than boys (AOR: 0⋅57; 95 % CI 0⋅34, 0⋅94). Similarly, children who consumed BM (AOR: 1⋅97; 95 % CI 1⋅20, 3⋅24), who were anaemic (AOR: 2⋅27; 95 % CI 1⋅38, 3⋅72) and who drank unsafe water (AOR: 1⋅91; 95 % CI 1⋅19, 3⋅07) were more likely to be underweight than their counterparts. In conclusion, CaM consumption was associated with lower prevalence of stunting and underweight than BM. Promoting CaM in pastoralist areas may help to curb the high level of undernutrition.

## Introduction

Undernutrition is a major global health problem with estimates in 2017 of 821 million undernourished people^(1)^. Based on 2017 estimates of undernutrition among children aged <5 years, 151 million were stunted and 50 million were wasted^(1)^. Accordingly, nutrition-related factors, especially undernutrition, contributed to approximately 45 % of deaths in children aged <5 years. In sub-Saharan Africa (SSA), <5 years old child death remains the highest in the world^(2)^, and estimates in 2017 were that globally 15 000 children aged <5 years died every day^(3)^. Ethiopia was among the top five countries for a total number of <5 years old child deaths in 2018, according to a UNICEF report^(4)^. Moreover, the rates of all types of growth failure (i.e. stunting, wasting and underweight) in Ethiopia remain a public health concern^(5)^. The Ethiopian Somali region has lower stunting and relatively higher underweight and wasting compared with other regional states of Ethiopia^(5,6)^.

Childhood undernutrition has developmental, economic, social and medical impacts which are serious and lasting, for individuals and their families, for communities and countries^(7)^. Furthermore, lower academic performance in school and cognitive deficit and poor economic development in adulthood are associated with childhood undernutrition^(8)^. An immediate cause of childhood undernutrition is an inadequate intake of nutritious food and a high burden of disease, with numerous underlying contributors. These include food insecurity, poor child feeding practices, lack of improved sanitation and lack of access to quality health and nutrition services, all of which are linked to socio-economic status and sociocultural contexts^(8,9)^.

In pastoral areas of Ethiopia, there has been a growing interest in milk production and consumption; consumers are looking for foodstuffs containing milk and milk products as important contributors to a healthy and balanced diet for children^(10,11)^. Cow and camel milk (CaM) are the most often consumed milk types for infants and young children; other types such as goat and sheep milk are consumed very rarely by children^(12,13)^. However, the price of unpasteurised and pasteurised milk is relatively expensive in Somali and Afar regions of Ethiopia as compared with other regional states because of greater demand and more extensive use of milk in their meals^(14,15)^.

In Ethiopian pastoral Somalis, cereal and cereal products are common in children's diets^(15)^. Moreover, milk is a staple of the traditional diet of Somali pastoralist children. The milk is obtained from either bovine (BM) or camel (CaM), but CaM is the preferred product that can be used fresh (*ma'an*), slightly sour (*suusa*) or sour (*garoor*)^(13)^. According to a 2010/11 report, Ethiopia produces about 4 billion litres of milk per year where 83 % is BM followed by 17 % from CaM^(16)^, but per capita consumption is very low, estimated at about 20 litres. CaM can supply required nutrients for child nutrition and eliminate the allergy complications sometimes caused by certain protein fractions in BM^(17)^. The composition of CaM *vs.* BM (in g/100 mL) includes: protein (3⋅1 *vs.* 3⋅5), fat (3⋅5 *vs.* 3⋅5), ash (0⋅79 *vs.* 0⋅76) and total solid (11⋅9 *vs.* 12⋅5), calcium (114 *vs.* 115 mg/100 mL) and iron (0⋅30 *vs.* 0⋅04 mg/100 mL).

Furthermore, compared with BM, CaM lacks β-lactoglobulin. However, CaM contains lactoferrin, a protein also found in human breast milk^(18–20)^. These milk nutrients have potential to contribute in explicit ways to the growth process and linear growth of children^(19)^.

According to several studies, CaM is different from BM in its composition and also has nutraceutical properties. Nutraceuticals found in CaM include insulin-like peptides, lysozymes, lactoferrin and antioxidative vitamins^(20,21)^ with antidiabetic, hypoallergenic, antihypertensive, anticancer and antimicrobial effects with substantial benefits for human health as a functional food^(17,20,22,23)^. To date, there are several studies conducted on BM consumption and childhood undernutrition in developed and developing countries^(24)^; however, to our understanding, there is a gap regarding the associations between CaM or BM consumption and growth failures in Ethiopia and elsewhere. Therefore, the main aim of the present study was to compare the prevalence of growth failures between CaM and BM consumers. Secondly, we investigated associations between CaM or BM consumption and other predictors of growth failures in rural pastoral districts of Somali, Ethiopia.

## Methods and materials

### Study design, setting and sampling

A community-based comparative cross-sectional study was carried out among 388 pre-schoolers, aged 24–59 months from November to December 2018. For the present study, two pastoral districts, namely Degahabour and Harorays, were randomly selected from high camel and cattle population districts, respectively.

The sample size required for the study was estimated to be 369 children by using a single population proportion formula based on 60 % anaemia among <5 years old children in a previous study^(25)^, 95 % confidence level and 5 % margin of error. Adding 10 % non-response rate, the final sample size was 406 including both CaM- and BM-consuming pre-schoolers. The sample was equally allocated to the two strata by the source of milk being consumed by children.

A multistage sampling technique was employed. The districts were selected randomly from those with camel or cattle populations. Then the study kebeles (smallest administrative units) were selected randomly from each district. Households in each kebele were stratified by the source of milk given to their children (CaM *vs.* BM). The presence of a lactating camel or lactating cow in the household was obtained from health extension workers and was taken as a marker to stratify the households. From each stratum, a child aged 24–59 months was selected randomly at each household. If more than one eligible child was present in the household, a child was selected by the lottery method. Researchers were not blind to the source of milk consumed by the child, because interviews and measurements were done at the household level.

### Data collection

Socio-demographic, economic, water, sanitation and hygiene, as well as milk feeding practices, were collected by individual interviews of mothers by trained data collectors fluent in Af-Somali and English. The questionnaire was pre-tested in kebeles other than the study kebeles before the data collection. Additionally, mothers were asked if their child had consumed food from a list of seven food groups on the previous day from the time that the child awakened until the child slept. The food groups were (i) cereals, tubers or roots; (ii) meat, poultry and fish (flesh foods); (iii) milk and dairy products; (iv) eggs; (v) nuts/legumes; (vi) vitamin A-rich fruits or vegetables; (vii) other fruits and vegetables. These seven categories are among the food groups suggested for evaluating diets of children^(26)^.

### Blood biochemical assessments

A finger prick blood sample was collected from each child according to WHO guidelines^(27)^. Haemoglobin (Hb) concentration was measured using HemoCue (Hb 301 model) with regular calibration following manufacturer's instructions (HemoCue Ltd, Sheffield, UK). A child with an Hb value of <11 g/dLwas considered as anaemic^(28)^. The use of the HemoCue has been validated in field studies, and the coefficient of variation has been reported as 3⋅9 %^(29)^. All children diagnosed with anaemia were immediately referred to local health facilities for further treatment.

### Anthropometric measurements

The researcher and assistant conducted anthropometric measurements of each child at home. The height and weight measures were taken twice with bare feet and light clothes following standard procedures. Child age was obtained from a parental recall using an events calendar. Body weight was measured with a SECA Model 874 electronic digital weighing scale (Seca GmbH & Co KG, Hamburg, Germany) to the nearest 0⋅1 kg. The scale was calibrated at least twice a day against a standard weight. The height was measured in an erect position to the nearest 0⋅1 cm using a calibrated portable SECA stadiometer (Seca GmbH & Co KG, Hamburg, Germany). During measurement of the height, the head was positioned at the Frankfurt plane and the four body parts (heel, calf, buttocks and shoulder) touched the vertical stand of the stadiometer. Using the WHO growth standards^(30)^, weight-for-age, height-for-age and weight-for-height *z*-scores were determined using WHO Anthro version 3.1.0^(31)^ for children aged <5 years.

### Data analysis

Frequency distributions and confidence intervals (CIs) were calculated for child and household characteristics. Additionally, frequencies for these characteristics were calculated based on the type of milk consumed. Furthermore, frequencies for food group consumption and anthropometric indices also were calculated for the group as a whole as well as based on the type of milk consumed. Pearson's *χ*^2^ test was used to determine associations with the background characteristics, food group consumption and milk source. Independent sample *t*-tests identified significant differences in each of the anthropometric indices based on the type of milk consumed. For the analysis of factors predicting growth failure, the normality of continuous data was checked by the Kolmogorov–Smirnov test. The potential predictors for anthropometric measurements were identified by bivariate analysis with *P* < 0⋅05 for inclusion in the multivariable analysis model. Multicollinearity was checked using the variance inflation factor (VIF) statistic, with VIF > 10 as the indication of multicollinearity for the logistic regression model. A forward Wald stepwise model was developed, and odds ratios were used to evaluate factors associated with child growth. All statistical analyses were performed with SPSS version 20 (IBM, Armonk, NY, USA), and statistical significance was reported at *P* < 0⋅05.

## Results

The mean  (sd) age of the children was 38⋅5 (10⋅2) months. Slightly more than half of the study participants were boys. About two-thirds of children were fed breast milk beyond 2 years and more than nine in ten children were ever breastfed. Moreover, more than half of pre-schoolers consumed milk ≥4 times/week (Table 1).

**Table 1.** Percentage distribution of child and household characteristics among CaM- and BM-consuming pre-schoolers in Somali region, Ethiopia

Variables		All	CaM	BM	
		*N* 386–388	*N* 185	*N* 203	*P*
	*n*	% (95 % CI)	*n* (%)	*n* (%)	
Child characteristics					
Sex					
Boy	198	51⋅3 (46⋅1, 56)	95 (51⋅4)	103 (50⋅7)	0⋅90
Girl	188	48⋅7 (44, 53⋅9)	90 (48⋅6)	100 (49⋅3)	
Age (months)					
24–41	243	62⋅6 (58⋅0, 67⋅5)	114 (61⋅6)	129 (63⋅5)	0⋅69
41–59	145	37⋅4 (32⋅5, 42⋅0)	71 (38⋅4)	74 (36⋅5)	
Duration of breastfeeding					
<2 years	121	31⋅3 (27⋅2, 36⋅0)	68 (36⋅8)	54 (26⋅6)	0⋅03
≥2 years	265	68⋅7 (64⋅0, 72⋅8)	117 (63⋅2)	149 (73⋅4)	
Breastfed ever					
Yes	368	95⋅7 (93⋅3,97⋅4)	176 (95⋅1)	194 (95⋅6)	0⋅84
No	18	4⋅3 (2⋅6, 6⋅7)	9 (4⋅9)	9 (4⋅4)	
Milk consumption per week					
1–3	184	47⋅4 (42⋅5, 52⋅1)	105 (56⋅8)	79 (38⋅9)	≤0⋅001
≥4	204	52⋅6 (47⋅9, 57⋅5)	80 (43⋅2)	124 (61⋅1)	
Any child health problem in the last 2 weeks					
Yes	46	11⋅9 (8⋅8, 15⋅0)	32 (17⋅5)	14 (6⋅9)	0⋅001
No	340	88⋅1 (85⋅0, 91⋅2)	151 (82⋅5)	189 (93⋅1)	
Anaemia status					
Anaemic	232	59⋅8 (54⋅9, 64⋅4)	79 (42⋅7)	153 (75⋅4)	≤0⋅001
Non-anaemic	156	40⋅2 (35⋅6, 45⋅1)	106 (57⋅3)	50 (24⋅6)	
Household characteristics					
Maternal educational status					
No schooling	379	97⋅5 (96⋅1, 99⋅0)	179 (96⋅8)	200 (98⋅5)	0⋅25
Attended school	9	2⋅5 (1⋅0, 3⋅9)	6 (3⋅2)	3 (1⋅5)	
Maternal employment status					
Self-employed/home-worker	301	78⋅0 (73⋅8, 81⋅9)	139 (75⋅1)	164 (80⋅8)	0⋅18
Labour/non-employed	85	22⋅0 (18⋅1, 26⋅2)	46 (24⋅9)	39 (19⋅2)	
No. of children aged <5 years					
1–2	300	77⋅3 (72⋅9, 81⋅4)	137 (74⋅1)	163 (80⋅3)	0⋅14
>3	88	22⋅7 (18⋅6, 27⋅1)	48 (25⋅9)	40 (19⋅7)	
Total family size					
≤6	153	39⋅4 (35⋅1, 44⋅3)	66 (35⋅7)	87 (42⋅9)	0⋅15
≥7	235	60⋅6 (55⋅7, 64⋅9)	119 (64⋅3)	116 (57⋅1)	
Health care facility					
Modern health facility	327	84⋅7 (80⋅8, 88⋅1)	169 (91⋅4)	159 (78⋅3)	≤0⋅001
Traditional healers	59	15⋅3 (11⋅9, 19⋅2)	16 (8⋅6)	44 (21⋅7)	
Drinking water source					
Safe/improved	215	55⋅7 (50⋅5, 60⋅4)	142 (76⋅8)	74 (36⋅5)	≤0⋅001
Unsafe/unimproved	171	44⋅3 (39⋅6, 49⋅5)	43 (23⋅2)	129 (63⋅5)	
Time spent to fetch water					
Less than 1 h	258	66⋅8 (62⋅4, 71⋅8)	103 (55⋅7)	156 (76⋅8)	≤0⋅001
Greater than 1 h	128	33⋅2 (28⋅2, 37⋅6)	82 (44⋅3)	47 (23⋅2)	
Water drink in the household					
Untreated	354	91⋅2 (88⋅6, 94)	161 (87⋅0)	193 (95⋅1)	0⋅005
Treated^a^	34	8⋅8 (6⋅0, 11⋅4)	24 (13⋅0)	10 (4⋅9)	
Waste disposal					
Buried/compost/fertiliser	296	76⋅5 (72⋅2, 80⋅9)	139 (75⋅1)	158 (77⋅8)	0⋅53
Throw to roadside/farm	92	23⋅5 (19⋅1, 27⋅8)	46 (24⋅9)	45 (22⋅2)	
Presence of toilet/latrine in household					
Yes	148	38⋅3 (33⋅7, 43⋅3)	74 (40⋅0)	74 (36⋅5)	0⋅47
No	238	61⋅7 (56⋅7, 66⋅3)	111 (60⋅0)	129 (63⋅5)	
Hand washing practice					
Never wash	96	24⋅7 (20⋅6, 29⋅1)	48 (25⋅9)	48 (23⋅6)	0⋅60
Wash hands at key times^b^	292	75⋅3 (70⋅9, 79⋅4)	137 (74⋅1)	155 (76⋅4)	

a Physically or chemically treated.

b Wash hands after defaecation and cleaning child faeces or before cooking and breastfeeding.

Almost all mothers of the pre-schoolers had never attended school, more than three-fourths were self-employed/home-workers and 60⋅6 % had greater than or equal to seven persons in a household. About 61⋅7 % of children were living in households with no toilet/latrine, 44⋅3 % drank unsafe/unimproved water and 8⋅8 % drank untreated water. Moreover, nearly one-fourth of mothers never washed their hands after defaecation or before cooking and breastfeeding.

Substantially more than half of children in both CaM and BM strata were fed breast milk beyond 2 years of age. A higher proportion of BM consumers had milk four or more times in a week period than did CaM consumers. In total, 75 % of BM consumers but fewer than 45 % of CaM-consuming children were anaemic as presented in Table 1.

We also found that a significantly higher proportion of pre-schoolers who consumed either CaM or BM used modern health facilities than traditional health facilities. More children who consumed CaM than BM reported health problems in the last 2 weeks; however, more CaM-consuming children reported safe/improved water sources in the household than did BM-consuming children.

### Food group patterns among children consuming CaM and BM

In the present study, the major food sources for children in the pastoral districts were dairy products and grains, roots and tubers (Table 2). Very few (0⋅8 %) pre-schoolers consumed eggs, 16⋅5 % consumed flesh foods, 16⋅8 % consumed other fruits and vegetables and 22⋅4 % consumed vitamin A-rich fruits and vegetables. Only three CaM consumers and no BM-consuming pre-school children ate eggs in the present study. In addition, both CaM- and BM-consuming pre-school children had in general low flesh foods (i.e. meat, fish, poultry and liver/organ meat) and other fruits and vegetables. However, CaM-consuming children had significantly lower flesh foods and higher other fruit and vegetable intakes as compared with BM- consuming children in the 24 h preceding the day of our interview as presented in Table 2.

**Table 2.** Food group consumption patterns of pre-schoolers aged 24–59 months in rural pastoral districts of Somali, Ethiopia

Food group(s)	All (*N* 388)	CaM (*N* 185)	BM (*N* 203)	*P*
	*n* (%)	*n* (%)	*n* (%)	
Grains, roots and tubers				
Yes	385 (99⋅2)	185 (100)	200 (98⋅5)	0⋅09
No	3 (0⋅8)	0	3 (1⋅5)	
Legumes and nuts				
Yes	156 (40⋅2)	71 (38⋅4)	85 (41⋅9)	0⋅48
No	232 (59⋅8)	114 (61⋅6)	118 (58⋅1)	
Dairy products (milk, yoghurt and cheese)				
Yes	388 (100)	185 (100)	203 (100)	−
Flesh foods (meat, fish, poultry and liver/organ meat)				
Yes	64 (16⋅5)	21 (11⋅4)	43 (21⋅2)	0⋅01
No	324 (83⋅5)	164 (88⋅6)	160 (78⋅8)	
Eggs				
Yes	3 (0⋅8)	3 (1⋅6)	0	0⋅07
No	385 (99⋅2)	182 (98⋅4)	203 (100)	
Vitamin A-rich fruits and vegetables				
Yes	87 (22⋅4)	45 (24⋅3)	42 (20⋅7)	0⋅39
No	301 (77⋅6	140 (75⋅7)	161 (79⋅3)	
Other fruits and vegetables				
Yes	65 (16⋅8)	41 (22⋅2)	24 (11⋅8)	0⋅01
No	323 (83⋅2)	144 (77⋅8)	178 (88⋅2)	

### Anthropometric measures by milk source

Stunting, underweight and wasting prevalence in the rural districts of Somali are presented overall and by CaM or BM consumption strata in Table 3. Overall, 24⋅1 % (95 % CI 20⋅5, 28⋅3) of pre-schoolers were stunted, 34⋅8 % (95 % CI 29⋅9, 39⋅6) were wasted and 34⋅7 % (95 % CI 30⋅1, 39⋅9) were underweight. Moreover, a higher proportion of pre-schoolers consuming BM were stunted (72 *vs.* 28 %; *P* < 0⋅001) and underweight (70⋅1 *vs.* 29⋅9 %; *P* < 0⋅001) compared with CaM consumers. In contrast, the prevalence of wasting was more similar and not significantly different between BM and CaM consumers. In BM compared with CaM-consuming pre-schoolers, significantly higher severe stunting (76 *vs.* 24 %; *P* = 0⋅002), severe wasting (66 *vs.* 34 %; *P* = 0⋅048) and severe underweight (78 *vs.* 22 %; *P <* 0*⋅*001) were observed.

**Table 3.** Pre-school children aged 24–59 months – anthropometric measures by milk source in rural pastoral districts of Somali, Ethiopia

Characteristics	All			CaM		BM		*P*
	*N*	Mean/%	95 % CI	*N*	Mean/%	*N*	Mean/%	
Stunting								
HAZ, mean (sd)	386	−0⋅81 (1⋅73)	−0⋅99, −0⋅64	184	−0⋅36 (0⋅12)	202	−1⋅22 (1⋅64)	<0⋅001
Any stunting	93	24⋅1	20⋅5, 28⋅3	26	28⋅0	67	72⋅0	<0⋅001
Severe stunting	38	9⋅8	7⋅3, 12⋅5	9	23⋅7	29	76⋅3	0⋅002
Wasting								
WHZ, mean (sd)	385	−1⋅50 (1⋅48)	−1⋅65, −1⋅36	183	−1⋅37 (1⋅47)	202	−1⋅63 (1⋅49)	0⋅086
Any wasting	134	34⋅8	29⋅9, 39⋅6	58	43⋅3	76	56⋅7	0⋅223
Severe wasting	47	12⋅2	9⋅4, 15⋅1	16	34⋅0	31	66⋅0	0⋅048
Underweight								
WAZ, mean (sd)	386	−1⋅47 (1⋅28)	−1⋅59, −1⋅34	184	−1⋅12 (1⋅15)	202	−1⋅79 (1⋅30)	<0⋅001
Any underweight	134	34⋅7	30⋅1, 39⋅9	40	29⋅9	94	70⋅1	<0⋅001
Severe underweight	54	14⋅0	10⋅9, 17⋅9	12	22⋅2	42	77⋅8	<0⋅001

*Note*: CI; confidence interval; severe stunting: height-for-age *z*-score (HAZ) < −3 sd; any stunting: HAZ < −2 sd; severe underweight: weight-for-age *z*-score (WAZ) < −3 sd; any underweight: WAZ < −2 sd; severe wasting: weight-for-height *z*-score (WHZ) < −3 sd; any wasting: WHZ < −2 sd.

### Factors predicting childhood undernutrition

After adjusting with covariates in the multivariable logistic regression model, the odds of stunting were more likely to be higher among anaemic children [adjusted odds ratio (AOR): 4⋅22; 95 % CI 2⋅23, 7⋅98], and children who consumed BM (AOR: 2⋅10; 95 % CI 1⋅22, 3⋅61) compared with those children without anaemia and who consumed CaM. Being a girl reduced odds of stunting by 43 % compared with boys (Table 4).

**Table 4.** Factors associated with stunting, wasting and underweight in rural pastoral districts of Somali, Ethiopia

Characteristics	Predictor category	AOR	95 % CI	*P*
Stunting^a^				
Factors remaining in the model				
Anaemia status	Anaemic	4⋅22	2⋅23, 7⋅98	≤0⋅0001
Sex of child	Girl	0⋅57	0⋅34, 0⋅94	0⋅03
Milk source consumed	BM	2⋅10	1⋅22, 3⋅61	0⋅007
Underweight^b^				
Factors remaining in the model				
Milk source consumed	BM	1⋅97	1⋅20, 3⋅24	0⋅008
Anaemia status	Anaemic	2⋅27	1⋅38, 3⋅72	0⋅001
Drinking water source	Unsafe/unimproved water	1⋅91	1⋅19, 3⋅07	0⋅007
Wasting^c^				
Factors remaining in the model				
Drinking water source	Unsafe/unimproved water	1⋅93	1⋅25, 2⋅97	0⋅003
Water treatment used	Untreated	2⋅77	1⋅03, 7⋅44	0⋅04

a Factors tested in the model: drinking water source, anaemia, milk source, breastfed ever, health care facility, time spent to fetch water, age of child, sex of child and frequency of milk consumed per week.

b Factors tested in the model: water treatment in the household, drinking water source, health care facility, time spent to fetch water, monthly income, anaemia, milk frequency consumed per week, dietary diversity, milk source and sex of child.

c Factors tested in the model: drinking water source, anaemia, milk type, breastfed ever, health care facility, time spent to fetch water, age of child, sex of child and water treatment in the household

Similarly, the odds of underweight were more likely to be higher among anaemic children (AOR: 2⋅27; 95 % CI 1⋅38, 3⋅72), children who consumed BM (AOR: 1⋅97; 95 % CI 1⋅20, 3⋅24) and who drank unsafe water (AOR: 1⋅91; 95 % CI 1⋅19, 3⋅07) compared with children without anaemia who consumed CaM and drank safe water.

Wasting was significantly associated with drinking unsafe and untreated water in households (*P* = 0⋅003 and *P* = 0⋅04), respectively. Drinking unsafe water (AOR: 1⋅93; 95 % CI 1⋅25, 2⋅95) and drinking untreated water (AOR: 2⋅77; 95 % CI 1⋅03, 7⋅44) in the household increased odds of wasting by nearly twice and more than twice, respectively, compared with those who drank safe and treated water.

## Discussion

The overall growth failure of pre-schoolers was high in the present study with 24⋅1 % of pre-schoolers being stunted, 34⋅8 % were wasted and 34⋅7 % were underweight. Stunting was less than the national figure (38 %), while wasting and underweight were higher than the national rates of 10 and 24 %^(5)^, respectively. A higher prevalence of stunting was reported in some parts of Ethiopia such as Amhara^(32)^, Bule Hora district^(33)^ and Belesa district of northwest Ethiopia^(34)^ which ranged from 27⋅6 to 57⋅7 %; these higher numbers might suggest poor child care practices and lack of access to health facilities^(35)^. However, a lower prevalence of stunting was observed in Gambia (15⋅7 %) and Wolayta-Sodo, Southern Ethiopia (22⋅2 %) as compared with our present result^(36,37)^.

In the present study, wasting was threefold higher compared with several results reported in other parts of Ethiopia such as Amhara, Bule Hora district, Wolayta-Sodo, Southern Ethiopia, Belesa district, northwest Ethiopia and in Gambia^(32–34,36,37)^ that ranged from 5⋅5 to 16 %. Similarly, lower childhood underweight was reported as compared with the present study in several studies^(32,36,38)^. On the other hand, higher childhood underweight was found in a study in India^(39)^.

Milk is known to provide essential nutrients for growth during childhood, but available nutrients differ by milk sources. CaM has positive nutritional and health benefits compared with BM because of its easy digestion and absorption for children^(20,40)^. In our present study, children who consumed BM were more vulnerable to stunting and underweight as compared with CaM consumers. Several studies and reviews report that CaM has antimicrobial and antiparasitic factors as well as inflammation inhibitors that have beneficial effects to reduce childhood stunting and underweight^(21,22,40)^. Moreover, CaM has potential to reduce nutrition-related iron deficiency anaemia by providing ten times higher concentrations of iron and five times higher vitamin C as compared with BM^(20)^ which could contribute to the reduction of childhood stunting and underweight. Furthermore, CaM has higher concentrations of insulin-like growth factor-1 and growth-promoting nutrients like zinc and niacin relative to BM^(41)^.

In general milk consumption in childhood has long been assumed to be positively associated with growth. This association may be due to the nutritious content of milk such as high levels of proteins and micro- and macronutrients as well as high calcium and the insulin-like growth factor-1 that are of major relevance for children's development and growth^(11,19)^. In the present study, more than half of pre-schoolers consumed milk more than four times in a week which might support childhood growth. However, a significantly lower proportion of CaM-consuming pre-schoolers had milk ≥4 times/week compared with BM consumers. This might be an explanation for the higher intakes of the other fruit and vegetable food groups by the CaM consumers than BM consumers as presented in Table 2 of the present study.

Our present study showed that 9⋅8 % of pre-schoolers were severely stunted, 12⋅2 % were severely wasted and 14⋅0 % were severely underweight. The children who consumed CaM had a lower proportion of severe stunting, wasting and underweight than BM consumers. CaM's effect on the reduction of infections caused by parasites and pathogenic microorganisms could have contributed to these differences^(22,42,43)^.

Our present results showed that boy pre-schoolers were more stunted than girls. Sex differences in pre-school children's height status also have been reported in low-income countries such as Guatemala, Senegal and various SSA countries^(44–46)^. Perhaps boys are more vulnerable to health inequalities than their girl counterparts in the same age groups^(45)^. The study finding was also consistent with previous research that reported boy children were more vulnerable to stunting because they required comparatively more calories for growth and development^(8,47,48)^. One of the reasons for low caloric intake in children might be low socio-economic status, based on findings in Pakistan and SSA^(8,48)^. Similarly, a study conducted in Guatemalan indigenous children showing that boys required greater energy intakes than girls could also make them more likely to be vulnerable for stunting with low socio-economic status^(46)^.

The odds of childhood stunting significantly increased with anaemia as compared with non-anaemic children. The likelihood of increased childhood stunting due to anaemia is consistent with several studies from low- and middle-income countries such as Ethiopia, Gambia, Guinea and China^(36,49–51)^. Anaemia may lead to increased susceptibility to childhood stunting by decreasing food intake, and thus inadequate satisfaction of required iron from the diet might have negative impacts on children's growth^(52,53)^. Additionally, the risk of underweight was significantly higher among anaemic children compared with non-anaemic. This result was consistent with previous findings that showed an increase in underweight in children with anaemia^(54)^ and, thus, may be associated with poor intake of family food rich in iron and loss of appetite because of anaemia infection^(50)^.

The odds of childhood stunting and underweight significantly increased in BM-consuming as compared with CaM-consuming pre-schoolers. To our understanding, there has not been a comparative study of the effects of BM and CaM consumption on child growth, but some studies have shown that milk consumption could contribute to anaemia and stunting in young children^(50,55)^. However, reports have indicated that CaM has unique potential for nutraceutical and therapeutic activities against different conditions such as anaemia, parasitic infections and milk protein allergies related to β-lactalbumin as compared with BM^(17,21,40,41,56–59)^ that could reduce stunting and underweight among the pastoralist children. Additionally, CaM has been fed as a human milk substitute in some studies^(20)^ and has had antimicrobial effects^(42,60)^.

For pre-schoolers in the present study, the odds of being wasted or underweight were between two and three times higher among children who drank unsafe/unimproved/untreated water in their household. A systematic review of studies in SSA and a report based on Demographic Health Survey data in Guinea found similar results^(8,49)^ regardless of the milk type to be consumed by pre-schoolers. Unsafe and unimproved water is not free of pathogens and these could contribute to childhood illness, wasting and underweight. In sustainable development goal 2030, target 6⋅1 aims to ensure universal and equitable access to safe and affordable drinking water for all^(61)^; however, adequate safe and improved water sources for Somali rural pastoralist children remain limited and are immense challenges putting them at risk of loss of nutrients due to diarrheal diseases and contribute childhood undernutrition.

Among limitations, cross-sectional data were used for our present study analyses; thus, temporal relationships could not be characterised in the pathways from milk intake to child growth. Also, we could not differentiate causal relations on the outcomes. We did not demonstrate a mechanism by which CaM improved child growth compared with BM, but presented fact-based evidence about characteristics of CaM. We have not collected detailed child infection information. We suggest further characterisation of CaM and evaluation of its impact on the health and growth of children with well-designed clinical trials.

## Conclusion

Lower prevalence of stunting and underweight were observed among CaM-consuming compared with BM-consuming pre-schoolers. Severe growth failures of all types persisted among BM consumers. Both milk type and anaemia status predicted childhood stunting and underweight. Drinking water sources were significant predictors of both wasting and underweight of pre-schoolers. Being girls significantly reduced the odds of childhood stunting. Promoting CaM consumption for pre-schoolers in pastoralist and agro-pastoralist areas may be helpful to curb the high rates of undernutrition in the Somali region.
